# The “Dialogue” Between Central and Peripheral Immunity After Ischemic Stroke: Focus on Spleen

**DOI:** 10.3389/fimmu.2021.792522

**Published:** 2021-12-16

**Authors:** Hongchen Yu, Yichen Cai, Aiqin Zhong, Yunsha Zhang, Junping Zhang, Shixin Xu

**Affiliations:** ^1^ Medical Experiment Center, First Teaching Hospital of Tianjin University of Traditional Chinese Medicine, Tianjin, China; ^2^ Tianjin Key Laboratory of Translational Research of TCM Prescription and Syndrome, Tianjin, China; ^3^ Tianjin University of Traditional Chinese Medicine, Tianjin, China; ^4^ National Clinical Research Center for Chinese Medicine Acupuncture and Moxibustion, First Teaching Hospital of Tianjin University of Traditional Chinese Medicine, Tianjin, China; ^5^ School of Integrative Medicine, Tianjin University of Traditional Chinese, Tianjin, China

**Keywords:** ischemic stroke, spleen, neuroinflammation, brain-spleen crosstalk, immune response

## Abstract

The immune response generated by the body after the incidence of ischemic stroke, runs through the comprehensive process of aftermath. During this process of ischemic stroke, the central neuroinflammation and peripheral immune response seriously affect the prognosis of patients, which has been the focus of research in recent years. As this research scenario progressed, the “dialogue” between central nervous inflammation and peripheral immune response after ischemic stroke has become more closely related. It’s worth noting that the spleen, as an important peripheral immune organ, plays a pivotal role in this dialogue. Multiple mechanisms have previously been reported for brain-spleen crosstalk after ischemic stroke. Further, neuroinflammation in the brain can affect the peripheral immune state by activating/inhibiting spleen function. However, the activation of the peripheral immune inflammatory response can work reversibly in the spleen. It further affects intracerebral neuroinflammation through the injured blood-brain barrier. Therefore, paying close attention to the role of spleen as the pivot between central and peripheral immunity in ischemic stroke may help to provide a new target for immune intervention in the treatment of ischemic stroke. In the present review, we reviewed the important role of spleen in central neuroinflammation and peripheral immune response after ischemic stroke. We summarized the relevant studies and reports on spleen as the target of immune intervention which can provide new ideas for the clinical treatment of ischemic stroke.

## Introduction

Ischemic stroke (IS) accounts for about 80% of all cerebrovascular diseases, and it is characterized by a rapid progression, high rates of disability, death and recurrence. Notably, it is a major disease which causes death and disability in humans worldwide (1), and imposes a heavy financial burden on families and society (2). Previous studies show that the immune system is specifically involved in the whole development of IS. The important features involved in the pathophysiology of IS are related to neuroinflammation, changes in immune cells and migration of immune cells, and dysregulation of peripheral immunity (3). Strong inflammatory cascade in the brain is observed during the early stage of IS development. The peripheral immune organs are gradually involved in the overall disease process, the blood-brain barrier(BBB) begins to be disrupted and permeability is altered. The interaction of central and peripheral immunity exacerbates neurological and vascular damage, and the immune system goes into imbalance, which is associated with increased susceptibility and risk of complications, as well as a poor prognosis.

The spleen is a secondary lymphoid organ, which is also the largest immune organ in the body and plays an important role in both innate and adaptive immune responses (4). Numerous studies have confirmed that the spleen appears to be a reservoir of immune cells, storing a large number of immune cells. During the acute phase of IS, the spleen is significantly reduced in volume and dramatic changes in internal composition during the acute phase of IS, a process that establishes a critical connection to the ischemic brain. The multiple “alarm” signals raised by the injured brain are critical for the initiation of brain-spleen communication, and then the activation of the spleen in turn feeds some information back to the brain, thus affecting neuroinflammation. The state of peripheral immunosuppression after IS is also associated with dysregulation of the spleen (4–6). The spleen appears to play an important “pivotal” role in linking central and peripheral immunity during IS, although the mechanisms by which the spleen links central and peripheral immunity are still unclear. Nowadays, the use of the spleen as a potential therapeutic target for immunomodulation is gaining attention and has the potential to be an important breakthrough in the treatment of IS.

## Neuroinflammation in Ischemic Stroke

Generally, the process of secondary brain injury and neuronal death caused by cerebral ischemia leads to the activation of local immune cells, resulting in inflammation of brain tissue (7). The inflammatory response occurs hours after IS and develops over the following weeks. This leads to secondary brain injury, infarct expansion and dysfunction of secondary organ after stroke (8). Following cerebral ischemia-reperfusion, reactive oxygen species (ROS) start to cause initial disruption of the BBB through upregulation of inflammatory mediators and activation of matrix metalloproteinases (MMPs) (9), followed by destabilization of the central nervous tissue and the internal environment. The release of Damage-associated molecular patterns (DAMPS) and the contents of dead and necrotic neurons into the extracellular environment and the subsequent triggering of a strong innate immune response (10). As the BBB is disrupted, monocytes, neutrophils, and T cells migrate into the brain, further activating preexisting microglia and expanding infarcts in brain tissue. In addition, microglia and peripheral immune cells from the central nervous system (CNS), including blood-derived monocytes/macrophages, neutrophils, and lymphocytes are recruited to the ischemic cerebral hemisphere, which ultimately triggers a strong inflammatory response (11, 12). This inflammatory response activates the complement system, summoning immune cells to the intrathecal compartment. Moreover, neutrophils, monocytes and lymphocytes cross the blood-brain barrier and converge towards the site of injury. Once these cells reach at the site of injury, they are activated to secrete free radicals, pro-inflammatory cytokines, prostaglandins and other inflammatory mediators. This leads to the recruitment of more immune cells and microglia at the site of injury (13). Exosomes secreted by microglia and astrocytes cause damage to the vascular endothelium of the blood-brain barrier by storing and releasing inflammatory cytokines, and some anti-inflammatory factors secreted by both can weaken the inflammatory response, which is associated with neuronal remodeling (14, 15). Previous studies and increasing evidence showed that a large number of immune cells are involved not only in the neuroinflammatory process but also in the maintenance of CNS homeostasis (16), while further expansion of neuroinflammation accelerates the clearance of necrotic tissue and neuronal repair.

## Immunosuppression in Ischemic Stroke

Changes in the nature of the internal environment caused by central nervous inflammation inevitably cause a disruption in the balance of the immune system. Systemic immune downregulation is observed within hours after cerebral ischemia, with suppression of cellular immunity. Generally, it is evidenced by increased apoptosis or the development of cellular dysfunction (17), along with the suppression of multiple inflammatory factors including IL-10, IL-1β, TNF-α, and IL-6. This immunosuppressed state is known as stroke-induced immune depression syndrome (SIDS) (18). The main processes leading to immunosuppression is related to the change from the lymphocyte phenotype T-helper Th1 to Th2 phenotype, a decrease in lymphocyte and NK cells counts in the blood and spleen, and impaired defense mechanisms of neutrophils and monocytes (19). This process is associated with the activation of the hypothalamic-pituitary-adrenal axis and the sympathetic nervous system (SNS), with increased secretion of adrenocorticotropic hormones and catecholamines, which exerts immunosuppressive effects (20). The immediate clinical consequence of immunosuppression in stroke patients is related to increased susceptibility to stroke-associated infections, of which pneumonia and urinary tract infections are the most common forms. Both of these forms are reported with an incidence of 10% (21). Notably, after IS, the main sites of immunosuppression appear to be located at peripheral immune organs and immune tissues, which in turn affect central neuroinflammation, and ultimately drives the immune response of the body into a new phase.

## The Role of the Spleen in Ischemic Stroke

As a fact, the spleen is a storage site for immune cells (22) and also is a major disseminator of inflammatory cells in response to injury, which plays an important role in secondary neurological injury after IS. Previous studies have shown that the spleen can serve as a “communication hub” for various immune cells interactions, including T cells, B cells, macrophages/monocytes, and dendritic cells, to initiate adaptive immune responses (23). After the onset of IS, the spleen is the main source of these peripheral immune cells that gets recruited into the brain (24, 25). Activation of astrocytes and microglia promotes local inflammation and release of pro-inflammatory cytokines, which in turn leads to the recruitment of peripheral blood immune cells. Many of the immune cells in this process are derived from the spleen (26, 27).

**Figure 1:**
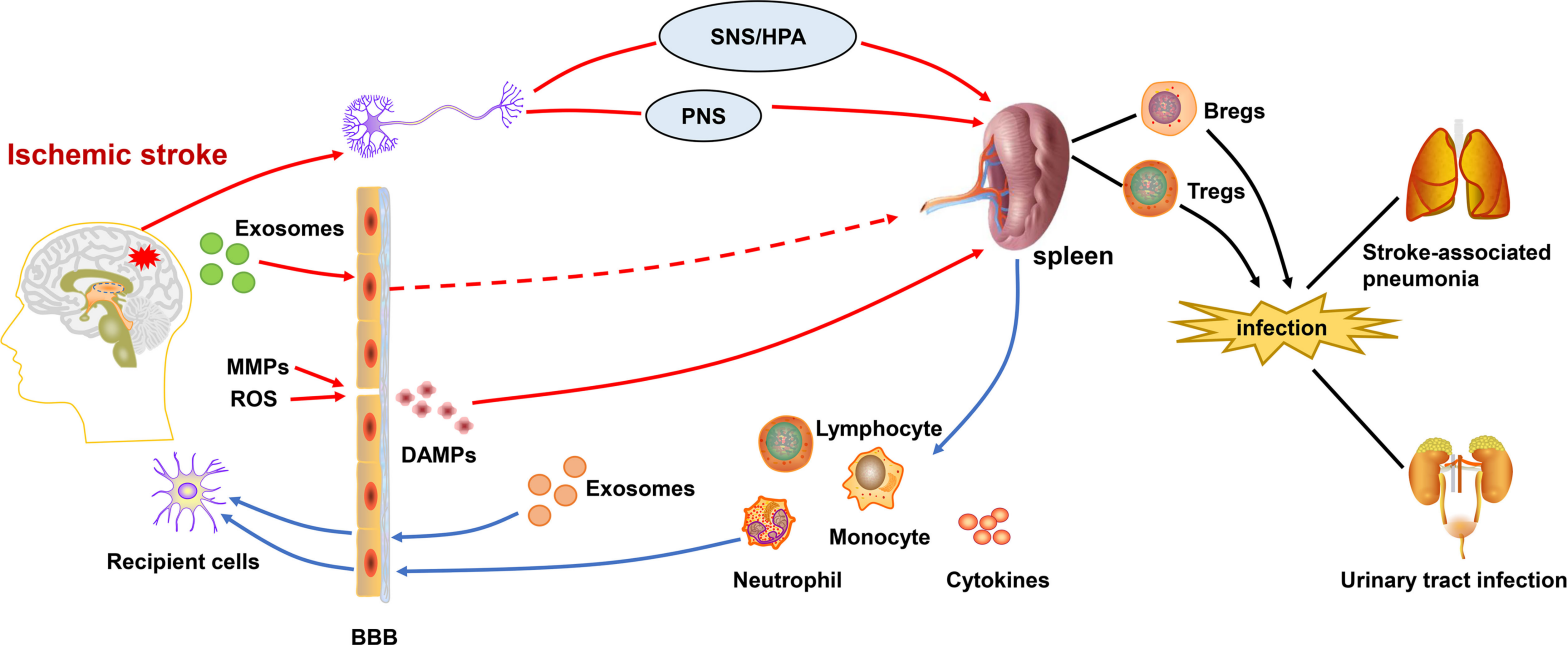
Central-peripheral/brain-spleen communication after ischemic stroke. After the onset of ischemic stroke, ROS and activated MMPs exacerbate the destruction of the BBB. DAMPs are released into the periphery, activating the immune system. Inflammatory signals from brain are transmitted to the spleen via SNS/HPA and PNS. Exosomes also play a role in linking central-peripheral immunity. The spleen is able to mobilize themigration of immune cells and therelease of cytokines, which affects the neuroinflammation in ischemic stroke. Correspondingly, the splenic response also leads to peripheral immunosuppression,which increases the risk ofstroke-associated pneumonia and urinary tract infections. BBB, blood-brain barrier; DAMPs, damage-associated molecular patterns; SNS/HPA, sympathetic nervous system/hypothalamus-pituitary-adrenaline axis; PNS, parasympathetic nervous system; MMPs, matrix metalloproteinases; ROS, reactive oxygen species.

Some experimental studies have shown that splenocytes in the injured hemisphere at 48 and 96 hours after middle cerebral artery occlusion(MCAO). For peripheral immunity after IS (24), the spleen’s own production of inflammatory factors also plays an immunomodulatory role. Atrophy of the spleen volume and reduction of lymphocytes is an important feature of immunosuppression (28), which are probably related to the massive migration and apoptosis of cells in the spleen and the shift in the expression of inflammatory factors. On the one hand, changes in peripheral immunity are governed by the brain, on the other hand, it also influences the brain’s own inflammatory response. While, the spleen plays a key role in the communication between central and peripheral immunity as shown in **Figure 1**.

## Central-Peripheral/Brain-Spleen Communication Mechanisms

### Sympathetic Nervous System/Hypothalamus-Pituitary-Adrenaline Axis

In the previous studies, various mechanisms have been reported of brain-spleen crosstalk after IS, in which the SNS and the hypothalamic-pituitary-adrenergic axis (HPA) are the predominant modes of communication. Generally, the spleen is sourced with the rich sympathetic supply, and is primarily innervated by sympathetic nerves. Stroke can induce enhanced sympathetic signals and induce splenic atrophy. The keys for effective brain splenic communication after stroke are related to the activation of the SNS, production of chemotactic cytokines, control of antigen presentation in the damaged brain, activation of the autonomic nervous system (ANS), release of CNS antigens and chemokine/chemokine receptor interactions (29).

The hypothalamic-pituitary-adrenal axis is stimulated by vagal afferent fibers, leading to the release of cortisol from the adrenal glands, which exerts anti-inflammatory effect. After IS the pro-inflammatory cytokines secreted by the spleen stimulate neurons located near the CRF neurons of the paraventricular nucleus of hypothalamus(PVN), and activating the HPA axis (30). Activation of the hypothalamic-pituitary-adrenal axis and the catecholaminergic axis can lead to splenic atrophy and a decrease in the number of peripheral NK cells by regulating the expression of cytokine signaling 3(SOCS3) (31). Splenic sympathetic nerves are stimulated by the vagus nerve. Norepinephrine released from the distal splenic nerve is linked to β2-adrenergic receptors in splenic lymphocytes that release acetylcholine. Acetylcholine(ACh) inhibits the release of TNFα from splenic macrophages *via* α-7-nicotinic acetylcholine receptors (30). Catecholamines such as epinephrine and norepinephrine are mediators of the SNS and are secreted when the SNS is activated. They inhibit Th1-lymphocyte activity and cellular immune responses through processes such as β-adrenergic receptor action of immune cells, and inhibition of IFN-γ production with stimulation of IL-10 production by immune cells (19).

Animal experiments showed that activation of α1-adrenergic receptors expressed in the rat spleen envelope causes splenic contraction (11), while, activation of β2-adrenergic receptors and glucocorticoid receptors (GCR) promoted immune cell apoptosis, reduced cell proliferation and increased cytokine production. Application of the β-adrenergic receptor blocker propranolol and the GCR blocker RU486 reversed splenic contraction by pharmacological inhibition of the SNS/HPA axis. This confirms the key role of SNS/HPA after IS (32). Treatment of MCAO mice with the β-blocker propranolol blocked catecholaminergic receptors and partially inhibited the activation of the SNS in these animals. The effects of this blockade in the circulation are related to an increase in spleen volume, an increase in IFN-γ levels and a decrease in IL-10 (33).

### Parasympathetic Nervous System

The parasympathetic nervous system (PNS) is another important mediator related to the brain’s regulation of the splenic and peripheral immune systems. The study carried out by Buijs et al. (34) showed that the spleen receives not only sympathetic but also parasympathetic inputs. Sympathetic input reaches the spleen through the arteries, whereas, parasympathetic input reaches the spleen through both ends of the spleen. the PNS modulates splenic immune responses through vagal activation and α7 nicotinic acetylcholine receptor (nAchR) stimulation, which may be related to the cholinergic anti-inflammatory pathway. This process is mediated by vagal efferent fibers that synapse to enteric neurons, which ultimately releases ACh at synaptic junctions along with macrophages. ACh binds to the α-7 nicotinic ACh receptors of these macrophages and inhibits the release of tumor necrosis TNF-α, thus exerting an anti-inflammatory effect (30). It has been shown that in an animal model of IS, activation of the PNS by intraperitoneal injection of α7nAchR agonist reduced caspase-3 cleavage in neurons, while it improved neural recovery and attenuated systemic neuroinflammation in rats (32). In fact, it is widely believed that the parasympathetic nervous system intervenes in the spleen in an indirect way by participating in the brain-spleen “dialogue”, and the corresponding mechanisms remain to be further elucidated.

### Other Potential Mechanisms

In addition to the SNS/HPA axis and the PNS, numerous previous studies reported potential mechanisms of brain-spleen communication. Using lipopolysaccharide (LPS) pretreatment as a method to induce ischemic tolerance in mice, the study carried out by Lidia et al. (35) observed production of neuroprotective monocytes in the spleen and that these monocytes transported to the brain and meninges and inhibited post-ischemic inflammation. This monocyte population has a potent neuroprotective capacity against experimental ischemic brain injury, mediated through the inhibition of meningeal inflammation. However, the exact underlaying molecular mechanism is not yet fully understood.

CNS-specific antigens can directly activate the peripheral immune system and produce an autoimmune response that enhances secondary brain injury. Further, these antigens can come into the contact with the peripheral immune system through the BBB or through CNS lymphatic drainage into the lymph nodes. CNS-specific antigens, such as microtubule-associated protein-2, n-methyl d-aspartate receptor subunit n-2a and myelin basic protein (MBP), have been identified in the palatine tonsils and cervical lymph nodes of acute IS patients. This suggests that the peripheral blood immune system may get exposed to CNS antigens through lymphatic connections (36, 37). In animal models of IS wind, brain-derived antigens were found to activate CD4+ and CD8+ T cells in cervical lymph nodes and spleen (38). Further, they shift peripheral blood immune responses to a Th1-like pattern, which is associated with worse neurological prognosis.

The systemic inflammatory response after stroke is mainly driven by cytokines, chemokines and stress hormones. Exosomes as an important intercellular communication mediator can cross the BBB and possess the potential to transfer brain-derived antigens to the periphery and activate/exacerbate the peripheral immune response after stroke. Also, exosomes could transfer pro-inflammation signals from the periphery to barin resident cells. It appears that exosomes act as a bridge between central and peripheral immunity after brain injury (8, 39–42).

## The Response of the Spleen in Central-Peripheral Immunity Dialogue

### Immune Cells and Cytokines Associated with the Spleen

The cellular composition of the spleen changes dramatically after IS, Specifically, significant alterations are observed in splenic function which increases the production of circulating pro-inflammatory cytokines, induces systemic T and B lymphocyte activation, helps peripheral immune cells and inflammatory mediators to invade the brain as **Table 1**, and exacerbate the local inflammatory response (35). One of the studies carried out by Dotson et al. (52) observed increase in the T-cell activation markers CD25 and CD122 in the spleen during the early stages of ischemic stroke. While the study carried out by Seifert et al. (24) used Carboxyfluorescein diacetate succinimide ester(CFSE) to follow splenocyte migration after MCAO and showed that splenocytes such as lymphocytes, monocytes, neutrophils and NK cells can enter the body circulation and migrate to the brain, where neutrophils lead to disruption of the blood-brain barrier by releasing matrix metalloproteinase 9 (MMP-9) (43). This ultimately resulted in leading to more leukocyte infiltration and worsening neuroinflammation. Nevertheless, previous studies have shown that the protective effect of splenectomy 2 weeks before stroke is associated with a decrease in the number of neutrophils in the area of injury (53). Activation of phagocytic cell population is probably associated with upregulation of genes related to phagocytosis and protein hydrolysis, while, increased expression of lysosome-related proteins in the spleen (45). The decrease in splenic immune cells, especially T- and B-lymphocytes, can persist for several months, leading to a persistent state of immunosuppression in stroke patients and increases the chances of infection (44). This process allows closer central-peripheral dialogue. Notably, despite lymphocytes in the spleen were predominantly reduced, the number of some specific cell populations such as Tregs and Bregs are increased after IS (46), both of which have potential neuroprotective effects. It is evident that the splenic response also has a bidirectional effect on the immune system.

**Table 1:** Immune cells and cytokines associated with the spleen.

Immune cells & Cytokines	Change of content in spleen	Effects on inflammation and immunity
Neutrophilic granulocyte	Decrease	Release of matrix metalloproteinase 9 (MMP-9) and consequent disruption of BBB integrity (24, 43)
T-lymphocyte and B-lymphocyte	Decrease	Leads to a persistent state of immunosuppression and increased susceptibility (44)
monocytes	Decrease	Aggravating brain damage and immunosuppressive effects (45)
Tregs	Increase	Balances the production of anti-inflammatory and pro-inflammatory factors and acts as a neuroprotective agent (46).
Bregs	Increase	suppress inflammatory responses, attenuate neurological damage and modulate immunity (47)
mononuclear phagocyte system	Decrease	Immunosuppressive effect and increased chance of infection (24)
NK	Decrease	Increased levels of IFN-γ, causing brain damage (24)
IL-1β	Increase	Exacerbates inflammation levels and nerve damage (48)
IL-6	Increase	Exacerbates inflammation levels and nerve damage (48)
TNFα	Increase	Upregulates inflammation levels and removes necrotic tissue (49)
IFN-γ	Decrease	Activates neutrophils, monocytes, macrophages and NK, and increases the secretion of IL-1, Il-6, and TNFα (4)
IL-10	Increase	Inhibits the inflammatory response (50)
IL-33	Increase	Regulation of splenic t-lymphocyte cellular immune response related (51)

Tab1, Tregs, Regulatory T cells; Bregs, Regulatory B cells; NK, natural killer cell; IL-1β,Interleukin-1β; IL-6, interleukin-6; TNFα, tumor necrosis factor-α; IFN-γ, interferon-γ; IL-10, interleukin-10; IL-33, interleukin-33.

Splenic glial cells are a transcriptionally distinct subtype of glial cells intermediate between immune cells and sympathetic axons, and are probably involved in axonal sheathing and communication with neural and immune cells. Splenic glial cells are capable of expressing receptors for neurotransmitters produced by sympathetic axons (adrenergic, purinergic and neuropeptide Y), as well as cytokines, chemokines and their receptors (54), which may communicate with immune cells in the spleen as an alternative pathway for central-peripheral connections. In addition, the spleen acts as an amplifier of myeloid-derived suppressor cell(MDSC) proliferation (55) and as a reservoir for MDSCs (55, 56). After 24 hours of cerebral ischemia, the number of splenic PMN-MDSCs increase. Cerebral ischemia rapidly induces the proliferation of splenic PMN-MDSCs without migrating to the brain, while accelerates the increase of PMN-MDSCs in the bone marrow and their migration to the ischemic brain (57).

As a large number of immune cells are activated, then immune cells in the spleen are subjected to secrete a large number of cytokines. These cytokines are generally located in the brain and include TNF-α, IL-6, monocyte chemotactic protein-1 (MCP-1), and IFN-γ (4). Many splenocyte-derived inflammatory cytokines and chemokines, such as IFN-γ and interferon-inducible protein-10 (IP-10), have been shown to be essential for stroke-induced neurodegeneration.CD147 is a cell surface glycoprotein that has recently been shown to be an important mediator of inflammatory and immune responses (58). One of the studies carried out by Rong Jin et al. (48) found that CD147 is an important mediator of inflammation in response to the brain ischemia, which is a key mediator of splenic inflammatory activation. Splenic CD147 expression was rapidly upregulated at 4h and 24 h after the onset of ischemia. αCD147 (an anti-CD147 antibody) suppressed the splenic inflammatory response induced by cerebral ischemia, as evidenced by a decrease in splenic cytokine (TNFα, IL-6, IL-1β) and MCP-1 expression at 4h and 24 h after ischemia. In addition, αCD147 abrogated ischemia-induced inflammatory activation of splenic monocytes/macrophages. This shows that the spleen influences the linkage of central and peripheral immune-related substances and that imbalances in central and peripheral immunity are inextricably linked to the spleen.

## Evidence From In Vivo Animal Experiments

Early animal studies have shown that the spleen shrinks significantly in the acute phase after MCAO, whereas, MCAO-induced reduction in spleen volume correlates with the degree of ischemic injury (42, 59). The loss of splenocytes seems to be associated with apoptosis of splenocytes and loss of splenic structure, with a decrease in B cells and an increase in Tregs. Removal of the spleen prior to middle cerebral artery occlusion (MCAO) reduces infarct volume and inflammatory response, decreases immune cell infiltration into the brain, possess a protective function for the brain, and does not increase the risk of post-stroke infection (53, 60). The study carried out by Yuanyuan Ran et al. (61) found that removal of the spleen not only reduced neurodegeneration caused by stroke but also reduced cellular degeneration in other tissues of the body exposed to ischemic conditions. Immune alterations following splenectomy include an increased ratio of IFN-γ to interleukin-10 and significantly higher absolute lymphocyte, CD4+ T cell and CD8+ T cell counts (50, 62). The spleen plays a key role in cytokine regulation, with significant reduction in levels of IL-12 and TNF-α in stroke splenectomized mice compared to splenectomized mice. Compared to male mice with a resected spleen, male mice with an intact spleen had shown significant increase in circulating CD11b+ monocytes, which secrete inflammatory cytokines such as IL-1β, IL-6, and TNFα, maintain inflammatory responses and lead to tissue-specific monocyte homing (49). However, delayed splenectomy does not provide long-term protection or improve long-term functional recovery in the ischemic brain. The acute neuroprotection achieved by early splenectomy immediately after stroke is also not sustained in the long term. Long-term disturbances in B- and T-cell composition after splenectomy may be associated with long-term lack of protection (61).

In addition to resection of the spleen, the study carried out by Ostrowski et al. (63) found that targeted splenic irradiation to blunt the splenic response in rats 3h or 4h after tMCAO significantly reduced the infarct size. Low doses of gamma radiation (Gy) induced apoptosis in the spleen without damaging the spleen or surrounding organs or causing immunosuppression or increased infection.

## Evidence From Clinical Cohorts

Recent clinical data suggests that spleen size in stroke patients is dynamic, i.e., it is observed with early contraction followed by re-expansion (15). One of the studies carried out by Farhaan et al. (64) with 158 concurrent cohort patients showed substantial reduction in spleen volume of approximately 40% patients after the stroke. In patients with splenic constriction, all cytokine levels were observed to be elevated, with significant differences in IFN-γ, IL-6, IL-10, IL-12, and IL-13. This was associated with higher National Institute of Health stroke scale(NIHSS) on admission and during hospitalization. Patients with spleen constriction had higher NIHSS at hospital visit compared to patients without spleen constriction. While, patients with spleen constriction had a significantly higher overall median NIHSS during hospitalization than patients without spleen constriction (65). National Institute of Health stroke scale also decreased as patients’ spleen size increased (6). The volume of the spleen also seems to be a predictor of the progression of the patient’s disease.

A study by Jiun-Nong Lin et al. (66) showed that splenic injury or splenectomy can significantly increase the risk of developing IS. Patients with splenic trauma had a 1.74-fold increased risk of stroke if they did not undergo splenectomy, and about 2.05-fold increased risk of stroke if they underwent splenectomy (67). The study carried out by Carles et al. (43) found that women exhibited smaller infarct volumes and fewer T cells after stroke positively correlated with serum estradiol levels, and this loss of ovarian hormones in older women was associated with an increase in stroke and splenic constriction. Not only the spleen becomes smaller in size after IS, but interferon IFNγ levels are also observed to be increased in subjects with splenic constriction. IFN-γ is one of the main mediators released from the spleen in post-stroke neurodegeneration, which may serve as a biomarker of stroke outcome or a target for the treatment of stroke in certain subgroups of subjects (68). One of the studies carried out by Matthew et al. (69) found that patients with damaged spleen appeared to have a higher hypercoagulable state that instigates approximately 48 hours after injury and persists for at least 5 days. This phenomenon may be related to the effect of the spleen on coagulation factors and may also stem from the effect of the spleen on vascular inflammation, the exact mechanism of which is unclear, but it does increase the risk of complications.

## Spleen as a Potential Therapeutic Target

In recent years it has been discovered that some therapeutic approaches can modulate the immune response by acting directly or indirectly on the spleen, and the spleen has received increasing attention as a potential therapeutic target in ischemic stroke as **Table 2**.

**Table 2:** Spleen as a potential therapeutic target.

Treatment	Treatment techniques and drugs	Potential targets
Stem Cell Therapy	Inject human umbilical cord blood (HUCB) cells (68)	Immune cells and cytokines from the spleen
	Intravenous administration of human bone marrow stem cells (hBMSC) (28)	Spleen
	Multifunctional adult progenitor cells treated (70)	Spleen
Targeting Tregs and Bregs	resveratrol pretreatment (71)	Tregs in the spleen
	IL-2/IL-2Ab treatment (72)	Tregs in the spleen
	IL-33 pretreatment (51)	Tregs in the spleen
	Lipopolysaccharide pretreatment (47)	Bregs in the spleen
Blocking nerve conduction	Propranolol (59)	Splenic sympathetic nerve
	Carvedilol (59)	Spleen
	Agmatine (73)	Splenic sympathetic nerve
	Sodium butyrate(NaB) (74)	IGF-1 in the spleen
Targeting protein and receptors	SYK inhibitors (75)	Spleen
	Recombinant T-cell receptor ligand treatment (52, 76)	Recombinant T-cell receptors in spleen
	Bexarotene (77)	N2-type neutrophils in spleen
New Treatment Technologies	RIPostC (78)	Spleen
	Selective intravascular cooling (SEC) (44)	Spleen

## Stem Cell Therapy

A large number of studies in recent years have demonstrated the positive effect of stem cell transplantation on immune regulation after IS. Since the spleen is the main destination of lymphatic drainage, the cerebral lymphatic system may serve as an effective pathway for brain-spleen stem cell migration. Human umbilical cord blood (HUCB) cells have been shown to reduce infarction in rats after pMCAO, which resulted in protective effect on gray and white matter, and restoration of spleen size 48 hours after pMCAO (68). HUCB cells can alter the immune cell profile and cytokine production in the spleen by moving from the injection site (42), which in turn can reduce the number of infiltrating immune cells in the ischemic area, and inhibit the increase of inflammatory cytokines (79). In addition, HUCB cells preserve spleen weight (80), which can be helpful in the recovery from ischemic stroke. Intravenous administration of human bone marrow stem cells (hBMSC) preferentially migrated to the spleen and suppressed systemic inflammation. This is supported by the findings for the therapeutic potential of targeting peripheral inflammatory responses *via* the spleen to eliminate neuroinflammation (28).

Multifunctional adult progenitor cells (MAPC), derived from human pluripotent adult stem cells from bone marrow, have been shown to exert effective immunomodulatory effects in a variety of disease models. The study carried out by Bing Yang et al. (70) found that MAPC-treated splenocytes led to increased levels of IL-10 and decreased levels of pro-inflammatory cytokines such as IL-1β and TNF-α, by a mechanism that may be related to the blockage of cell transport to the brain was blocked. Indeed, the spleen is a key target for MAPC to modulate immune responses and promote recovery after stroke, however, it is not the only target for MAPC to exert therapeutic effects in the brain.

## Targeting Tregs and Bregs

With the activation of peripheral immunity, Tregs related with the spleen are able to balance the production of anti-inflammatory and pro-inflammatory factors through multiple pathways, driving the immune system to restore homeostasis. A variety of drugs that target the spleen may ultimately act by acting on regulatory T cells in the spleen. One of the studies reported that resveratrol pretreatment enhanced the production of T regulatory cells in the spleen of MCAO rats, which partially contributed to the protective effect on the ischemic brain (71). The study carried out by Haiyue Zhang et al. (72) found that treatment with IL-2/IL-2 antibodies complex can significantly increase the number of CD39+ Tregs and CD73+ Tregs in blood, spleen and lymph nodes, which exerted a neuroprotective effect against cerebral ischemia IL-33 is a novel cytokine of the IL-1 family with a protective effect against ischemic brain injury. The IL-33 pretreatment reduced the splenic IFN-γ production and increased IL-4, IL-10 and the transforming growth factor-β(TGF-β) secretion 24 h after MCAO. The protective mechanism of IL-33 may be related to the regulation of splenic immune response by promoting Tregs response. Therefore, IL-33 may be considered a candidate for treating human stroke via modulating the peripheral immune system after stroke (51).

Like Tregs, Bregs are also known to suppress inflammatory responses, attenuate neurological damage and modulate immunity. One of the studies carried out by Zhe Wang et al. (47) found that lipopolysaccharide pretreatment increased splenic regulatory B cell levels in mice after acute ischemia/reperfusion, which attenuated the inflammatory response in the spleen, mainly in the form of increased numbers of splenic follicular B cells and marginal zone B cells in mice. Recent studies suggest that the site of regulatory B-cell action is primarily observed in the spleen, and whether these protective B-cells are released from the spleen after stroke requires further study, which probably possess positive implications for exploring the spleen as a potential therapeutic target.

## Interventions On Brain-Spleen Crosstalk

Since the spleen is innervated by the SNS/HPA and influenced by the parasympathetic nervous system, some drugs may modulate immunity by affecting brain-spleen crosstalk. Studies have shown that treatment of MCAO mice with the β-blocker propranolol blocks catecholaminergic receptors and partially inhibits SNS activation in these animals, and this blockade is reflected in increased IFN-γ levels; decreased IL-10 and increased splenic volume (59). Carvedilol is an all-adrenergic blocker that reduces infarct size and prevents splenic contraction in rats with pMCAO (59), but the exact mechanism of action is not fully understood. Agmatine, a sympathetic neurolytic agent, can indirectly target the spleen and reduce infarct size (73). Ajmo et al. (59) blocked α1 receptors with pyrazosin or butanediol in animal experiments could block splenic contraction, while propranolol did not block contraction. Carvedilol also reduced infarct size. Sodium butyrate (NaB) is a histone deacetylase (HDAC) inhibitor, which can increases histone acetylation levels and promotes non-specific and specific immune system function. NaB was shown to be effective in reducing infarct size after MCAO in female rats by a mechanism that may be related to the ability to increase IGF-1 (a known neuroprotective agent). The distribution of this molecule in the spleen and its expression in serum and splenic peripheral tissues in the acute late phase of stroke (74).

## Targeting Spleen-Related Protein and Receptors

There are drugs that act on proteins or receptors related with the spleen to exert immunomodulatory effects on ischemic stroke. Splenic tyrosine kinase (SYK) is a non-receptor tyrosine kinase, mainly found in the spleen, thymus and other organs. It is expressed in a variety of cells and mainly associated with inflammatory processes. Oral administration of SYK inhibitors, protected mice from arterial thrombosis and thrombotic brain infarction. Mice lacking SYK in platelets were specifically protected against arterial thrombosis and IS, however, their hemostatic function was not altered (75). Recombinant T-cell receptors (RTLs), a class of partially major histocompatibility complex (MHC) class II molecules, are widely present in the brain and spleen of MCAO mice at 96 h post-stroke. Recombinant T-cell receptor ligand (RTL) treatment reduced infarct size by 50% in tMCAO mice and reduced the recruitment of immune cells to the brain, preventing a reduction in spleen volume (52, 76). A study carried out by Michelangelo et al. (77) found that the application of the agent retinoid X receptor (RXR) agonist bexarotene increased N2-type neutrophils in the ipsilateral hemisphere and spleen of mice with temporary middle cerebral artery occlusion to slow down central and peripheral inflammation and exert neuroprotective effects. This drug can be effectively used in the acute treatment of IS, with the spleen highlighted as a potential therapeutic target.

## New Treatment Technologies

In recent years with the advancement of new medical technologies, several novel therapeutic modalities have been advanced in the treatment of IS. Distal ischemic post-treatment (RIPC) is an interesting treatment modality that provides protection when applied immediately after ischemic injury to distal non-vital organs induced ischemia (81). This technique is widely used in the prevention and treatment of cardiovascular and cerebrovascular diseases. Previous studies have shown the protective effect of distal limb ischemic post-treatment (RIPostC) against transient focal cerebral ischemia-reperfusion injury. RIPostC can act on the spleen to reduce the level of B and NK cells in the spleen and increase non-inflammatory monocytes in the blood (78) to reduce the damage of inflammatory effects. Selective intravascular cooling (SEC) is the local cooling down of the brain while maintaining body temperature by infusion of cold saline through the intra-arterial arteries. One of the studies found that in IS SEC can upregulate splenic IL-10 expression, exerting anti-inflammatory effects and improving the peripheral inflammatory response (44).

## Discussion

Immune system imbalance after ischemic stroke is influenced by both central neuroinflammation and peripheral immune suppression. Activation of the immune system occurs from the center to the periphery and then throughout the body. The activated peripheral immunity in turn affects the center. Notably, CNS inflammation is not entirely harmful, and acute central inflammation after IS has long been considered “neuroprotective” (46, 82). As an example, activated microglia and CNS-specific T cells contribute to the maintenance of neurogenesis and spatial learning capacity in the adult brain. By investigating the sources and mechanisms of neuroinflammation, particularly the role of splenic-mediated inflammatory responses, identifying new cell death pathways and innovative therapeutic targets to interrupt this inflammatory process could provide avenues to ameliorate cell death secondary to stroke and traumatic brain injury. Severe systemic immunosuppression due to cerebral ischemia leading to infectious complications is a major cause of death in patients with IS (21). It has also been shown that the macrolide antibiotic azithromycin can act on the spleen and reduce splenic contraction to counteract immunosuppression (83), however, the observation of clinical data has not been carried out.

## Summary

In conclusion, the spleen occupies a unique position in the immune system and the immune response of the spleen is an indispensable link in the central-peripheral dialogue in ischemic stroke. Using the spleen as a target for immune modulation may open up new ideas for the treatment of ischemic stroke.

## Author Contributions

HY and YC drafted the article and performed the literature research. SX putted forward the idea of the article and critically revised the work. AZ, YZ and JZ reviewed and edited the article. All authors contributed to the article and approved the submitted version.

## Funding

This study was supported by the National Natural Science Foundation of China (No. 81774059) and the Tianjin Natural Science Foundation (No. 19JCZDJC37100).

## Conflict of Interest

The authors declare that the research was conducted in the absence of any commercial or financial relationships that could be construed as a potential conflict of interest.

## Publisher’s Note

All claims expressed in this article are solely those of the authors and do not necessarily represent those of their affiliated organizations, or those of the publisher, the editors and the reviewers. Any product that may be evaluated in this article, or claim that may be made by its manufacturer, is not guaranteed or endorsed by the publisher.

## Abbreviations

IS, ischemic stroke; BBB, blood-brain barrier; DAMPS, damage-associated molecular patterns; CNS, central nervous system; MMPs, matrix metalloproteinases; ROS, reactive oxygen species; SIDS, stroke-induced immune depression syndrome; SNS, sympathetic nervous system; MCAO, middle cerebral artery occlusion; ANS, autonomic nervous system; PVN, paraventricular nucleus of hypothalamus; SOCS3, cytokine signaling 3; ACh, acetylcholine; GCR, glucocorticoid receptors; IFN, interferon; PNS, parasympathetic nervous system; nAchR, nicotinic acetylcholine receptor; TNF-α, tumor necrosis factor-α; LPS, lipopolysaccharide; HPA, hypothalamic-pituitary-adrenergic axis; CFSE, carboxyfluorescein diacetate succinimide ester; MDSC, myeloid-derived suppressor cell; MCP-1, monocyte chemotactic protein-1; IP-10, interferon-inducible protein-10; Gy, gamma radiation; NIHSS, National Institute of Health stroke scale; HUCB, human umbilical cord blood; hBMSC, human bone marrow stem cells; MAPC, multifunctional adult progenitor cells; TGF-β, transforming growth factor-β; NaB, sodium butyrate; HDAC, histone deacetylase; SYK, splenic tyrosine kinase; RTLs, recombinant T-cell receptors; MHC, major histocompatibility complex; RXR, agent retinoid X receptor; SEC, selective intravascular cooling; MBP, myelin basic protein; Tregs, regulatory T cells; Bregs, regulatory B cells; NK, natural killer cell.
